# Evaluation of Two Veterinary Oscillometric Noninvasive Blood Pressure (NIBP) Measurement Devices (petMAP Graphic II and High-Definition Oscillometry) in Dogs

**DOI:** 10.3390/vetsci12040349

**Published:** 2025-04-09

**Authors:** Hanna Walter, Sabine B. R. Kästner, Thomas Amon, Julia M. A. Tünsmeyer

**Affiliations:** 1 Small Animal Clinic, University of Veterinary Medicine Hannover, 30559 Hannover, Germany; 2 Department of Veterinary Clinical Sciences, Clinical Pathology and Clinical Pathophysiology, Justus-Liebig-University, 35392 Gießen, Germany

**Keywords:** hypotension, high-definition oscillometry, vasoconstriction, hypertension

## Abstract

Noninvasive blood pressure (NIBP) device performance in dogs may be influenced by extreme pressures and altered systemic vascular resistance (SVR). This study evaluated the agreement of two NIBP devices (HDO and petMAP) with invasive blood pressure (IBP) measurements, compliance with hypertension consensus statement criteria, and their trending ability (TA) across varying blood pressure and SVR ranges in awake and anesthetized dogs. Seven healthy Beagles were studied, with IBP recorded from the dorsal metatarsal artery and NIBP cuffs placed randomly on the front limb, hind limb, or base of the tail. Cardiac output was determined by thermodilution, and the systemic vascular resistance index (SVRI) was calculated by a standard formula. Bland–Altman, concordance rate, and polar plot analyses were used for statistical analysis. A total of 752 and 640 paired measurements were obtained for HDO and petMAP, respectively. Both devices showed good agreement with IBP for mean arterial pressure (MAP) at low blood pressure and the SVRI. At high blood pressure and the SVRI, agreement weakened, with substantial underestimation of systolic arterial pressure (SAP). Both devices demonstrated moderate to good TA for MAP and SAP. Overall, the best agreement was observed for MAP at a low SVRI, while agreement was moderate at hypertension (petMAP) and a high SVRI (petMAP, HDO).

## Simple Summary

Reliable and valid blood pressure measurements are essential in many clinical situations. Hypo- or hypertension can have a great impact on vital organs. Unfortunately, extreme pressure ranges may negatively influence the reliability of noninvasive blood pressure monitors; particularly, anesthetic drugs affecting the vascular tone seem to have a negative impact. This study tested two veterinary oscillometric blood pressure devices, to see how well they matched invasive blood pressure measurements at different states of blood pressure and vascular tone, respectively. Seven healthy Beagle dogs were studied while awake and under anesthesia. The results showed that both devices became less accurate when blood pressure was high but were particularly underestimating the highest blood pressure values. Despite this, both devices were able to detect lower pressures and to track overall blood pressure trends reasonably well. When blood pressure is high, veterinarians should be cautious, as readings may not be accurate. This study highlights the importance of understanding the limitations of noninvasive blood pressure measurements in different situations for clinical decision making.

## 1. Introduction

Reliable blood pressure (BP) measurements are of utmost importance in veterinary medicine as undetected BP abnormalities can have serious consequences for vital organs. In anesthetized patients, measurement of BP is crucial since various sedatives and anesthetics have a major impact on BP, leading to hypo- or hypertension. Only if measured BP values are accurate and valid can clinicians make appropriate therapeutic decisions. Invasive blood pressure (IBP) is currently considered the clinical gold standard for BP measurements, as it is the most accurate and direct continuous BP measurement technique [1,2]. However, the drawbacks are technical difficulties especially in small and awake animals, like the risks of arterial catheterization, material costs, and invasiveness associated with painfulness for animals [3,4,5]. The accuracy of conventional oscillometry has been reported to be limited, especially in small animals and in hypo- and hypertensive ranges [6,7,8]. The oscillometric method measures the pressure oscillations transferred to the cuff during reoccurrence of blood flow in the artery. The maximal pressure amplitude oscillations occur at mean arterial pressure and are easily detected and measured. Common oscillometric devices all measure MAP by this technique, whereas SAP and DAP are based on different, device-specific algorithms often taken from human data [9], which explains the variable and limited accuracy of SAP and DAP. Modern measurement methods, like high-definition oscillometry (HDO), claim to be more accurate, e.g., at high pulse rates or extreme pressure ranges [10]. Drugs, which increase systemic vascular resistance like alpha_2_-agonists, might have a negative impact on the accuracy of NIBP devices [11,12,13]. Several veterinary studies investigated the agreement of different noninvasive blood pressure (NIBP) devices with IBP at different BP ranges. However, the influence of vascular tone and SVR on the performance of modern NIBP has not been assessed. Our hypothesis was that (1) MAP has good agreement with iMAP over all pressure ranges with both devices, (2) high systemic vascular resistance impairs derivation of DAP and SAP by oscillometry, and (3) both oscillometric devices are able to trend iMAP changes.

Therefore, the aim was to investigate the agreement of two modern veterinary oscillometric BP devices with IBP at low, normal, and high BP and SVRI ranges in dogs and their associated trending ability.

## 2. Materials and Methods

Seven beagle dogs aged 19 to 21 months and weighing 8.6 to 15.1 kg were included in the study, which was approved by the local animal welfare committee (protocol number 33.12-42502-04-16/2151). Prior to the start of the study, dogs were assessed as healthy based on physical examination, serum biochemistry, CBC, and echocardiography. Animals were cared for according to principles outlined in the NIH Guide for the Care and Use of Laboratory Animals. After completion of the study, all dogs were rehomed. The animal number was based on the required animals for a concurrent non-interfering study [14], and no a priori power analysis specific for the blood pressure study was performed.

Beagle dogs were exclusively used in this and the parallelly conducted experimental study because Beagles are a widely used breed in research due to their availability, standardized size, and consistent physiological characteristics, which help minimize variability and enhance the reproducibility of results.

The study was conducted in a randomized, complete crossover, experimental design.

### 2.1. Instrumentation

Following aseptic preparation, a 22-gauge catheter was placed in a cephalic vein of the dogs for fluid administration and drug delivery. Anesthesia was induced via facemask with sevoflurane in oxygen (8 Vol. % in 100% O_2_, flow rate: 3 Lmin). After achievement of a sufficient anesthetic depth and topical anesthesia of the larynx, orotracheal intubation was performed and animals were immediately connected to a small animal anesthesia machine via a circle breathing system (Fabius^®^ Trio, Drägerwerk AG & Co. KGaA, Luebeck, Germany). Anesthesia was maintained with sevoflurane in oxygen, dosed to achieve surgical tolerance, defined by loss of palpebral reflex, muscle relaxation, and immobility. Standard anesthetic monitoring included sidestream capnography, pulse oximetry, lead II ECG, inspiratory and end-expiratory gas concentrations, and body temperature, with the temperature sensor positioned in the nasal cavity (S/5^TM^ Collect, Datex-Ohmeda Devision Instrumentarium Corp., Helsinki, Finland). Dogs were covered with a warm air-filled blanket (Bair Hugger Modell 505; Arizant Healthcare, Goodyear, AZ, USA) to maintain body temperature between 37 °C and 38.5 °C. For IBP measurement, the skin over a dorsal pedal artery was aseptically prepared before cannulation of the artery by the Seldinger technique (CareflowTM, 2.5 French 60 mm, Argon Critical Care Systems Singapore Pe. Ltd., Singapore). Arterial catheters were connected to precalibrated electronic pressure transducers (Safedraw^TM^, pressure transducer set, Argon Critical Care Systems Pte. Ltd., Singapore) via non-compliant fluid-filled extension lines and systems were regularly flushed with heparinized saline (2 IU/mL) to prevent blood clot formation. All measured pressures (ABP, CVP, PAOP) were checked against an aneroid manometer (Boso Manometer; Bosch + Sohn, Jungingen, Germany) for the first measurements.

Pressure transducers were positioned and zeroed to ambient pressure at the level of the aortic root. The iSAP, iMAP, and iDAP were continuously measured, displayed on a multiparameter monitor, and recorded (S/5^TM^ Collect, Datex-Ohmeda Devision Instrumentarium Corp., Helsinki, Finland). Arterial pressure waveforms were evaluated by visual inspection and regularly performing fast flush tests to detect over- or underdamping. Visual inspections of IBP curves after fast flush tests did not reveal excessive over- or underdamping at any time during the analyzed measurements. For cardiac output (CO) measurements, the skin over a jugular vein was aseptically prepared and an 8 French catheter introducer (BD Exacta; Argon Critical Care Systems Pte. Ltd., Singapore) was placed in the jugular vein using the Seldinger technique. A balloon-tipped pulmonary artery thermodilution catheter (CriticathTM, 7 French, 110 cm, Argon Critical Care Systems Pte. Ltd., Singapore) was then advanced into the jugular vein via the introducer port until the distal lumen was located in the pulmonary artery. Correct positioning was guided by observation of characteristic pressure waveforms and confirmed by thoracic radiographs.

### 2.2. Data Recording and Analysis

For HDO and petMAP measurements, the device-specific cuffs were used, and cuff sizes were chosen as recommended by the manufacturers. For HDO, this was in all dogs and all three measurement locations the medium-sized cuff, recommended for dogs with a body weight of 8–15 kg. For the petMAP monitor, the size of cuff was chosen as where the index line of the cuff best fitted in the “optimum zone” as recommended by the manufacturer, resulting in cuff sizes 4.5–5.5 for the front legs, 4.0–4.5 at the hind legs, and 4.0 at the base of the tail, respectively. All cuffs were randomly placed over a common palmar digital artery, dorsal metatarsal artery, or the medial coccygeal artery and then rotated clockwise after each measurement cycle, in order to gain the same number of measurements with each device in each of the three locations. For each data comparison, a mean of six consecutive noninvasive measurements was calculated and compared to the mean of the invasive measurements from a dorsal pedal artery from the corresponding 6 min period for each device separately. All blood pressure measurements were taken in lateral recumbency with all cuffs positioned approximately at the level of the heart and with minimal fixation in awake animals, to avoid stress-induced effects on the cardiovascular system as much as possible. Ten CO measurements were performed altogether per dog and trial day, with the thermodilution technique. For each CO determination, thermodilution measurements were taken with a 5 mL bolus of iced 5% dextrose until 3 measurements within 10% deviation of each other were obtained or until a maximum of five bolus injections was reached. The three closest values were chosen for calculating the arithmetic mean as the representative CO and the SVRI was calculated afterwards according to a standard formula [15].

In sevoflurane anesthesia hypertension (iMAP > 120 mmHg) [16] and hypotension (iMAP < 60 mmHg) [16] were induced by administration of dopamine (10–18 µg kg^−1^ min^−1^ IV) or increasing the inspired fraction of sevoflurane, respectively. After stabilization of the hemodynamic state for at least 5 min, a CO measurement was taken at hypotension and hypertension, respectively. Dogs recovered from sevoflurane anesthesia after complete instrumentation and induced hypertension and hypotension. At least three hours after complete recovery from anesthesia, CO and SVRI baseline measurements were taken in the awake animals. As part of a non-interfering study [14,17], the dogs were randomly assigned to four different anesthetic protocols blinded to the investigators and including an alpha_2_ agonist, during which further CO, SVRI, and paired ABP measurements were performed. For full details, see publications of the concurrent study.

Therefore, blood pressure data were recorded in awake and anesthetized dogs and those were not differentiated for analysis. Blood pressure ranges for normotension (>60 mmHg; <120 mmHg), hypertension (>120 mmHg), and hypotension (<60 mmHg) were based on iMAP. Due to ethical reasons, the upper limit for hypertension was determined at 180 mmHg and the lower limit for hypotension at 40 mmHg, respectively.

A normal SVRI range (1523–1928 dynes/sec/cm^5^/m^2^) was determined by calculating the 99% confidence interval of SVRI baseline values, which were taken in awake animals. SVRI above or below this value were categorized as high or low, respectively. Oscillometric BP measurement data were analyzed via visual inspection of acquired deflation curves of the measured oscillations. Data were categorized into good, moderate, or poor measurements performed by the same person (HW) according to the manufacturer’s specifications. Poor measurements were excluded from statistical analysis. Inclusion criteria for statistical analysis were a set of BP measurements with a minimum of three consecutive obtained values. Criteria to characterize the data were device-dependent:

For the HDO, data characterized as “good” had a linear deflation rate, a distinct bell-shaped curve of the measured oscillations, and no artefacts. If there was no distinct bell-shaped curve or if there were minimal fluctuations in the linear deflation rate, the measurement was characterized as “moderate”. The data were characterized as “poor” if there were measurement errors, artefacts that biased the linear deflate rate moderately, irregular deflation pressures, and hereby a curve instead of a linear deflation function or a missing bell-shaped curve.

Measurement data obtained with the PetMAP device characterized as “good” had an evenly gradual pressure deflation rate and oscillation amplitudes presented as a bell-shaped curve consisting of individual bars. “Moderate” measurements had an unevenly shaped bell curve, but the curve shape was still preserved, and the deflation rate was evenly gradual. “Poor” measurements had an uneven gradual deflation rate and the oscillation amplitudes either did not depict a curve any longer or individual bars were completely missing, or individual bars were extremely high (outliers).

### 2.3. Statistical Analysis

Bland–Altman method of agreement between methods of measurement with multiple observations per individual was used for statistical comparison of NIBP and IBP measurements [18]. Mean differences (bias), one standard deviation (SD), and 95% confidence intervals (limits of agreement, LoA) of the difference were calculated by subtracting IBP values from corresponding NIBP values as described by BLAND and ALTMAN (2007) [18]. A positive bias reflected an overestimation by oscillometric devices and a negative bias an underestimation of IBP. Bland–Altman plots of differences in measurements against the mean of each pair of readings were prepared (GraphPad Prism 8.0, Graph-Pad Software, Inc., San Diego, CA, USA). Percentages of NIBP measurements, which lay within 10 mmHg and 20 mmHg of the reference method, were calculated.

Polar plot methodology [19] for inspection of TA of both oscillometric devices was performed with MedCalc software (version 23.2.1). Differences in consecutive measurements for each device and mean of differences in investigated NIBP and IBP methods were determined to gain the mean variation in ΔSAP or ΔMAP or ΔDAP. As described by CRITCHLEY et al. (2011) [19,20,21,22], data were converted to angles. Variation in BP was depicted as radiant. Since centrally located data points represent minor variations in BP with a high random error, an exclusion zone of 20% was included in the polar plots to eliminate those. Concordance rates, which demonstrate percentages of data points that lie within the desired ±30°, were calculated.

## 3. Results

Of 756 measurement attempts with each device, 752 (99.5%) and 640 (84.7%) paired measurements could be obtained with HDO and petMAP, respectively. All dogs tolerated the attachment of BP cuffs and BP measurements well.

With both devices, the best agreement was detected for MAP at low BP and SVRI ranges. At a high SVRI (HDO and petMAP) and hypertension (only petMAP), agreement was moderate. Considering SAP and DAP at hypertension and a high SVRI, the devices showed weak agreement. Under these cardiovascular conditions, both devices massively underestimated blood pressures, particularly iSAP. At normotension and a normal SVRI, HDO achieved good agreement for MAP, underestimating IBP. In these cardiovascular states, petMAP overestimated iMAP, resulting in an overall moderate agreement at normotension and good agreement at a normal SVRI. Table 1 demonstrates the number of obtained, paired measurements, bias, SD, LoA, and percentages of NIBP measurements lying within 10 and 20 mmHg of the reference method for SAP and DAP at the different investigated cardiovascular ranges.

**Figure 1:**
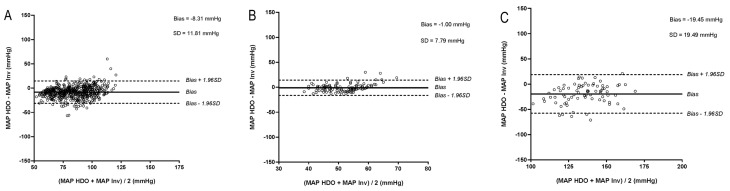

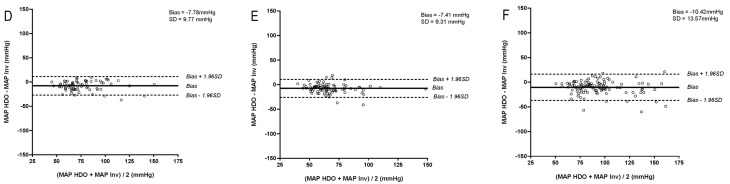
(HDO): Bland–Altman plots of agreement between invasively measured mean arterial blood pressure (iMAP) and mean arterial blood pressure obtained by high-definition oscillometry (MAP HDO) at (**A**) normotension (iMAP 60–120 mmHg), (**B**) hypotension (iMAP < 60 mmHg), (**C**) hypertension (iMAP > 120 mmHg, (**D**) SVRI in reference (1523–1928 dynes/s/cm^5^/m^2^), (**E**) SVRI low (<1523 dynes/s/cm^5^/m^2^), (**F**) SVRI high (>1928 dynes/s/cm^5^/m^2^). The solid line indicates the mean bias and the dashed lines the upper and lower limits of agreement.

**Figure 2:**
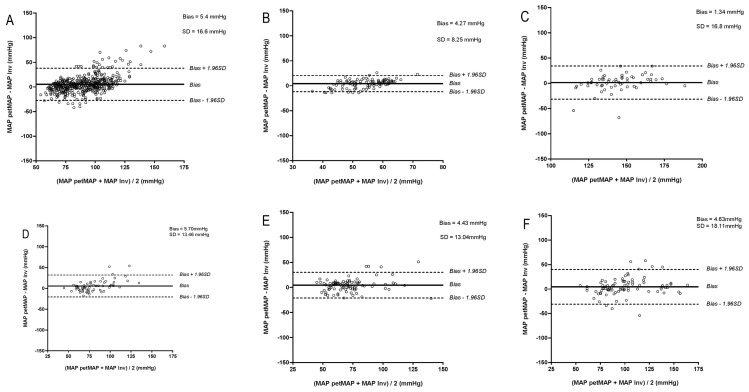
(petMAP): Bland–Altman plots of agreement between invasively measured mean arterial blood pressure (iMAP) and mean arterial blood pressure obtained by petMAP graphic II (MAP petMAP) at (**A**) normotension (MAP inv 60–120 mmHg), (**B**) hypotension (iMAP < 60 mmHg), (**C**) hypertension (iMAP > 120 mmHg, (**D**) SVRI in reference (1523–1928 dynes/s/cm^5^/m^2^), (**E**) SVRI low (<1523 dynes/s/cm^5^/m^2^), (**F**) SVRI high (>1928 dynes/s/cm^5^/m^2^). The solid line indicates the mean bias and the dashed lines the upper and lower limits of agreement.

At hypertension (average hypertensive iMAP: HDO: 146 mmHg, petMAP: 143 mmHg), the SVRI was high at the same time in merely 17.7% and 17.9% for HDO and petMAP, respectively. At a low SVRI, BP was simultaneously hypotensive (average hypotensive iMAP: both devices: 54 mmHg) in merely 33.7% and 33.3% for HDO and petMAP, respectively. Bland–Altman plots for assessing agreement between IBP and NIBP measurements of MAP at different cardiovascular ranges including bias and SD are presented in Figure 1 and Figure 2.

Table 2 demonstrates the percentages of HDO and petMAP measurements, which ranged within 10 mmHg and 20 mmHg of the reference method for MAP at the investigated cardiovascular ranges. Table 3 shows overall agreement of both devices with the reference method according to the ACVIM guidelines.

**Figure 3:**
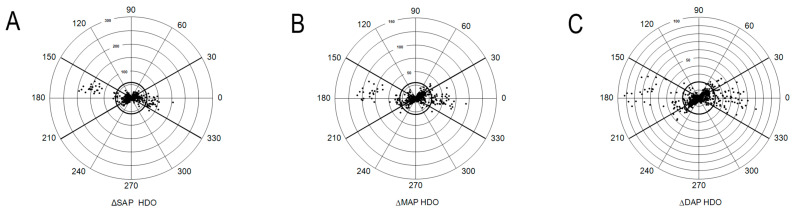
(HDO) Trending ability of the high-definition oscillometry (HDO) device compared to invasive blood pressure measurement is illustrated using a polar plot. Key advantage of a polar plot is that it visually represents how well the new method tracks changes in the reference method over time, rather than just comparing absolute values at single time points. Polar plots evaluate whether the new method accurately follows trends (i.e., increases and decreases) in the reference measurements. Each data point represents the difference between consecutive blood pressure measurements obtained by the two methods. The distance of a data point from the center indicates the mean difference in (**A**) systolic arterial pressure (ΔSAP), (**B**) mean arterial pressure (ΔMAP), and (**C**) diastolic arterial pressure (ΔDAP) in mmHg. The off-axis angle (0° radius) shows how well the HDO measurements align with the invasive reference method—points closer to 0° indicate better agreement. The bold circle in the center represents the exclusion zone of 20%, meaning data points within this area were not considered in the trending analysis.

**Figure 4:**
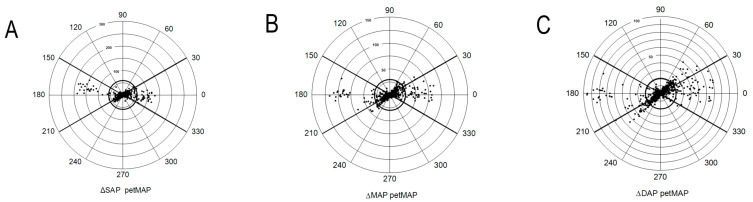
(petMAP) Trending ability of the petMAP graphic II device (petMAP) depicted as polar plot. For further explanation of a polar plot, see above legend of Figure 3. The distance of data points from the center of the plot represents the mean difference in (**A**) ΔSAP, (**B**) ΔMAP, (**C**) ΔDAP in mmHg. The off-axis angle (0° radius) depicts the agreement of the petMAP with the invasive reference method. The bold circle in the center of the plot indicates the utilized exclusion zone of 20%.

Good TA for SAP was detected with both devices. Moderate TA considering MAP was achieved by HDO. Polar plots for assessing the TA of oscillometric devices with concordance rates for SAP, MAP, and DAP for each device separately are presented in Figure 3 and Figure 4. After using a 20% exclusion zone, a total of 60, 87, and 126 and 55, 92, and 159 data points for SAP, MAP, and DAP were analyzed for HDO and petMAP, respectively.

## 4. Discussion

Overall, both oscillometric devices performed best in measuring MAP over the range of blood pressures with moderate to good agreement with iMAP, and our primary hypothesis is partially fulfilled. Both hypertension and a high SVRI impaired the derivation of DAP and SAP by oscillometry; however, both devices were able to trend changes in iSAP. Different studies show that extreme BP ranges have a significant impact on the accuracy of NIBP measurement devices [20,21,22]. Several studies assume the SVRI as possibly limiting or biasing the factor of BP measurements, especially if it is elevated [11,12]. This is a common problem in small animal anesthesia, due to the widespread use of α_2_-agonists [13] and can also occur in very anxious or fractious animals.

Therefore, we hypothesized that extreme SVRI ranges would result in profound inaccuracies, which was mainly confirmed by our results.

### 4.1. Impact of SVRI on NIBP Devices

In particular, extreme increases in the SVRI have a large impact on the measurement accuracy of NIBP devices and if all criteria of the ACVIM guidelines [23] are considered, none of the investigated devices gained an acceptable agreement with IBP for all three blood pressure ranges. The weakest agreement with IBP was detected at a high SVRI with both devices. Possible explanations could be increased vascular wall tension leading to impaired transfer of pressure oscillations to the air cuff, an incomplete bell-shaped oscillation curve if the occlusion pressure is not high enough, or the oscillation formation is impeded or minimized by contracted vascular walls. Comparing the results of Bland–Altman analysis at a high SVRI with those at hypertension, it is noticeable that with both devices, bias and SD are higher at hypertension. This is particularly true for SAP. In contrast to our hypothesis, hypertension seemed to have a greater impact overall on the measurement accuracy of the devices than a high SVRI. However, it needs to be considered that the SVRI influences BP, limiting the ability to differentiate the results of a high SVRI from hypertension. In addition, the number of measurements with high BP and a high SVRI were different, which is a limitation of our study and could have an impact on the results. The SVRI was categorized as “high” in this study, based on the calculation of the 99% confidence interval of the individual SVRI baseline values; therefore, it was not always extremely high. In addition, only 17–18% of the measurements obtained at hypertensive states were combined with an increased SVRI. This is because the SVRI could only be calculated afterwards, and MAP was the command variable during the measurements. In retrospect, it might have been beneficial to choose a more potent vasoconstrictor like noradrenaline instead of dopamine, to artificially induce hypertension concurrently with a high SVRI during the instrumentation phase. On the other hand, the idea for this study originated from the clinical observation that oscillometric blood pressure measurement reliability is strongly influenced by the perianesthetic use of alpha_2_-agonists, particularly given as CRI, and this was investigated during the second anesthesia phase of the study.

The theory that a high SVRI causes the damping of oscillations is supported by the underestimation of all pressures by HDO and of iSAP by petMAP. However, petMAP overestimates iMAP and iDAP. The results at a high SVRI for both devices are in general not convincing. Here, petMAP over- and HDO underestimates IBP and the very wide LoA must be considered.

In addition, a low SVRI has a significant impact on the accuracy of measurements, again with MAP being the parameter with the best agreement. A possible explanation for the weak agreement with iSAP and iDAP measured by petMAP is the underlying technique, where MAP is measured as a point of maximal pressure oscillations, whereas SAP and DAP are calculated based on device-specific algorithms. Yet, HDO, which, according to the manufacturer, measures all three parameters in microsecond cycle and real-time analysis [24], cannot gain good agreement with iSAP and iDAP, either. At normotension and a normal SVRI, the agreement of the investigated devices with iMAP is comparable. When comparing Bland–Altmann graphs at a low SVRI with those at hypotensive ranges, a significant deterioration in measurement accuracy and therefore agreement with IBP at a low SVRI can be observed. This is noticeable for both devices for iSAP and iDAP. At low SVRI ranges, both devices achieve good agreement with iMAP according to ACVIM guidelines and have comparable results.

### 4.2. Agreement in Normotensive State

In our study, the petMAP device underestimates SAP and overestimates MAP and DAP at normotension and shows a moderate agreement with iMAP, moderate to weak agreement with iDAP, and weak agreement with SAP. Comparable weak agreements considering SAP and DAP are found by SHIH et al. (2010) in their study in anesthetized dogs [25]. However, all parameters are considerably underestimated. As in our study, ACIERNO et al. (2013) found an underestimation of SAP and an overestimation of MAP and DAP compared to invasively measured BP in anesthetized dogs [20]. However, the authors conclude an overall weak agreement of the device. In contrast to this is the study of VACHON et al. (2014), where BP is measured with the petMAP device in awake and anesthetized dogs at normotension and shows a good agreement with invasively measured BP and the fulfilment of all ACVIM validation criteria [26]. Interestingly, all parameters are underestimated in anesthesia, while in awake animals, SAP is under- and MAP and DAP are overestimated. As the described studies show, SAP and DAP measured with the petMAP device often are not in agreement with the invasively measured SAP and DAP. However, for MAP, in most studies, an acceptable agreement could be detected. A reason for the differences in the results could be the utilized devices. Even though all studies use a petMAP device from the Ramsey Medical company, different device models (petMAP, petMAP graphic, petMAP graphic II) are utilized, which could be an explanation for the different outcomes.

Our study, the HDO device showed a good agreement with invasively measured MAP; however, SAP and DAP were massively underestimated, resulting in a weak agreement. These results are in accordance with the results of WERNICK et al. (2010), considering the agreement of the device with invasively measured BP in the A. metatarsalis dorsalis using fluid-filled arterial catheters [27]. In their study, in anesthetized dogs, invasively measured BP is over- instead of underestimated at normotension. The BP range at which measurements were conducted is a difference between the two studies (our study: 61 mmHg–119 mmHg (iMAP); WERNICK et al., 2010: 45 mmHg–96 mmHg (iMAP)), which could be a possible explanation why measurements were over- and not underestimated. Another important factor could be the great difference between the amount of measurement pairs (our study: n = 559; WERNICK et al. (2010): n = 63). In contrast to this are the results of SELISKAR et al. (2013), which show a good agreement with iMAP and iDAP in anesthetized, normotensive dogs [28]. Here, all criteria of the ACVIM are fulfilled.

### 4.3. Agreement in Hypotensive State

In our study, petMAP achieves good agreement with iSAP, iMAP, and iDAP at hypotension. For clinical use, it is a disadvantage that iMAP is overestimated, since relevant hypotension might remain unnoticed. This is similar to the results of other studies, which also reveal an overestimation of all BP parameters at hypotension. The studies investigated the agreement of petMAP with IBP measured with fluid-filled transducer systems in metatarsal arteries in anesthetized dogs. However, both studies detect a weaker agreement with IBP compared to the results of this study [20,25]. SHIH et al. (2010) [25] withdraw 40% of the dogs’ blood volume to gain stable hypotension with iMAP values ≤ 40 mmHg. Agreement of petMAP with iSAP, iMAP, and iDAP is poor at hypotension. The used technique for artificially lowering BP by reducing blood volume is an essential difference compared to our study. A physiologic reaction to hypotension due to blood loss is vasoconstriction. This is, as the results of our study at high SVRI ranges show, associated with a weaker agreement of the petMAP with IBP. In contrast, iso- and sevoflurane, which were used to induce hypotension in our study, cause vasodilation and a reduced SVRI, which is associated with better agreement at least considering iMAP and iDAP. Considering petMAP, the presented study is to our knowledge the first study in which BP ranges were separately analyzed.

The HDO device predominantly shows good agreement at hypotension in our study. Compared to our results, RYSNIK et al. (2013) [29], which measured IBP with a catheter placed in the dorsal pedal artery, gain similar bias and SD values. The study defines hypotension differently (iMAP < 70 mmHg) and iMAP and iDAP are overestimated instead of underestimated.

### 4.4. Agreement in Hypertensive State

In our study, petMAP achieves weak agreement with iSAP and moderate agreement with iMAP and iDAP at hypertension. Studies investigating the agreement of the petMAP device at hypertension are lacking in the literature.

In our study, HDO underestimates hypertensive ranges and the weakest agreement is obtained with iSAP. Likewise, MAP and DAP attain a high SD and therefore poor agreement with IBP. ACIERNO et al. (2010) [30] recognize a tendency of HDO to underestimate IBP at iSAP values ≥ 125 mmHg in anesthetized cats. In contrast, WERNICK et al. (2010) [27] report an increasing overestimation with increasing pressure in anesthetized dogs; however, hypertensive ranges are not reached. The results of our study are in contrast to the results of RYSNIK et al. (2013) [29] in anesthetized, hypertensive dogs, since HDO can achieve good agreement with iMAP considering bias and SD. Similar to our results, all BP parameters are underestimated at iMAP values above 100 mmHg and no convincing results are obtained due to high bias and SD considering SAP and DAP. Investigations differ in their definitions of hypertension (presented study: iMAP > 120 mmHg, RYSNIK et al. (2013) [29]: iMAP > 100 mmHg), which could be an explanation for the greater agreement of MAP in the study of RYSNIK et al. (2013) [29].

### 4.5. Trending Ability of NIBP Devices

An important feature of blood pressure monitors is the ability to correctly detect BP trends. To evaluate TA, the polar plot methodology described by CRITCHLEY et al. (2011) [19] can be used. Originally, this method was used to investigate the ability to detect trends of CO monitors [19]. Human studies also utilized it for the evaluation of NIBP devices [31,32]. CRITCHLEY et al. (2011) [19] define good agreement of CO monitors if data points lie within ± 30°. Concordance rates, which demonstrate percentages of data points that lie within the desired ± 30°, ≥ 95%, represent a good trending ability, which is achieved with both devices for SAP. The concordance rate of petMAP (100%) is marginally better than that of HDO (98.3%). Concordance rates within 90–95% are considered as a marginal trending ability, which is obtained by HDO for MAP. Poor TA (concordance rate < 90%) is obtained with petMAP for MAP and DAP and with HDO for DAP. Our results are surprising, since the Bland–Altman analysis revealed the poorest agreement with iSAP for both devices. Since MAP is the originally measured parameter, it is difficult to explain why petMAP could not trend MAP. Real-time analysis in microsecond cycles of HDO, measuring all three pressures, seems to be advantageous. However, the exact localization of measurement points or the algorithms used for this purpose by the HDO are not accessible. Even if NIBP devices cannot gain precise BP values, the ability to trend BP makes their measurements valid for clinical use. In our study, this could be shown for both devices for SAP and for HDO additionally for MAP.

### 4.6. Limitations

There are some limitations to our study. For the evaluation of our results, true “gold standard” criteria are lacking in veterinary medicine. The ACVIM guideline criteria were designed for the identification of hypertension in dogs and cats [23]. As in our study, many scientists use these for the detection of hypotension in small animal studies [29,33,34]. For hypotension, however, the LoAs defined in the ACVIM criteria are far too wide from a clinical point of view. Techniques that are considered as the technical “gold standard” like transducer-tipped catheters or radio telemetry, which can measure BP in centrally located arteries, were not used in our study. It is controversial whether central or peripheral BP is the “true” BP. Fluid-filled transducer systems with the catheter located in peripheral arteries were used in the current study, since NIBP devices also measure at peripheral arteries and IBP should be measured at a similar distance of the artery to the heart. A contributing factor to weak agreement with IBP could be over- or underdamping of the invasive measurement system, particularly affecting SAP and DAP [1,35,36,37]. In our study, however, these factors were minimized by the use of short, non-compliant tubing and the elimination of visible air bubbles. In addition, the systems were regularly visually checked for curve resonance and potential damping with fast flush tests. However, a certain influence on the accuracy of the invasive measurements, used as the reference method in this study, cannot be excluded as the dynamic indices like the damping coefficient of the system were not calculated.

Another factor limiting the accuracy of invasive blood pressure measurement as a reference method in our study was the placement of the arterial catheter in the dorsal pedal artery. A recent study compared the agreement between blood pressure values obtained using an oscillometric NIBP device, with the cuff positioned at the mid-antebrachium, and invasive measurements from four different peripheral arteries. The study found the best agreement with invasive measurements from the median caudal artery. While MAP and DAP measurements from the dorsal pedal artery also showed good agreement, the SAP values were significantly higher at this site [38]. The authors suggested that the median caudal artery may provide more accurate measurements than the dorsal pedal artery, particularly for SAP, as it has fewer branches from the aorta, reducing the influence of wave reflection. Since these wave reflection effects may be even more pronounced in hypertensive states, this could explain the underestimation of SAP by both oscillometric devices in our study, especially during hypertension. However, this confounding effect may have been mitigated in our study, as the oscillometric cuffs were rotated between three different locations, including the hind limb. In contrast, the aforementioned study used only oscillometric measurements from the antebrachium as a reference.

As the SVRI could only be calculated afterwards, hypertension was the command variable during the measurements and only a limited number of high SVRI states was achieved as discussed above.

A limitation in the TA analysis was the choice of the exclusion zone, which was based on the reported standards of BP measurement variability [23]. However, this exclusion zone (20%) was not the same as in other published articles and if the recommended exclusion zone (15%) for CO monitors from CRITCHLEY et al. (2011) [19] would be applied, the results would show an even weaker TA for both devices.

## 5. Conclusions

In both devices, the best agreement was detected for MAP at low BP and SVRI ranges. When measuring blood pressure with these oscillometric devices, MAP should be considered to be the most accurate parameter and therefore be given the most clinical relevance. Good TA for SAP was detected with both devices. Moderate TA considering MAP was achieved by HDO.

## Figures and Tables

**Table 1:** Comparison of Bland–Altman analysis results at the different investigated cardiovascular ranges.

			BP			SVRI		
			↔	↓	↑	↔	↓	↑
HDO	n		559	116	77	74	92	113
	SAP	Bias (mmHg)	−23.8	−0.4	−65.1	−21.6	−14.4	−33.3
		SD (mmHg)	21.8	15.5	31.6	23.6	18.0	29.5
		±10 (%)	19	52.6	2.6	25.7	34.8	13.4
		±20 (%)	42.2	84.5	6.6	50	69.6	26.8
	DAP	Bias (mmHg)	−12.9	−9.6	−21.5	−13.7	−13.3	−14.4
		SD (mmHg)	12.0	7.0	20.4	10.7	8.3	13.5
		±10 (%)	37.7	51.7	35.1	35.1	35.9	31
		±20 (%)	75.7	94.8	53.2	77	80.4	75.2
petMAP	n		471	110	59	64	90	84
	SAP	Bias (mmHg)	−12.8	−0.3	−39.2	−10.2	−4.8	−21.1
		SD (mmHg)	20.8	11.9	28.3	19.5	16.0	25.6
		±10 (%)	31.8	56.4	10.3	35.9	47.8	23.8
		±20 (%)	59	92.7	20.7	60.9	78.9	47.6
	DAP	Bias (mmHg)	2.9	−1.0	−0.7	1.5	−0.4	2.2
		SD (mmHg)	19.5	10.0	16.7	16.3	15.6	20.9
		±10 (%)	53.3	74.5	62.7	54.7	61.1	47.6
		±20 (%)	79	93.6	89.8	84.4	82.2	76.2

Abbreviations: SVRI: systemic vascular resistance index; ↔: in reference range, ↓: low (MAP: <60 mmHg, SVRI: <1523 dynes/sec/cm^5^/m^2^), ↑: high (MAP: >120 mmHg, SVRI: >1928 dynes/sec/cm^5^/m^2^) range; n: number measurement sets per device; HDO: high-definition monitor MD^Pro^; petMAP: petMAP graphic II device; SD: standard deviation; SAP, DAP: systolic, diastolic arterial pressure.

**Table 2:** Percentage of HDO and petMAP measurements within 10 mmHg and 20 mmHg of the reference method for MAP.

		BP			SVRI		
		↔	↓	↑	↔	↓	↑
HDO	±10 (%)	55	87.9	28.6	66.2	65.2	51.3
	±20 (%)	86	98.3	52	89.2	94.6	79.6
petMAP	±10 (%)	60.1	74.6	66.1	57.2	67.8	61.9
	±20 (%)	85.1	97.3	84.8	90.6	90	81

Abbreviations: SVRI: systemic vascular resistance index; ↔: in reference range, ↓: low (MAP: <60 mmHg, SVRI: <1523 dynes/s/cm^5^/m^2^),↑: high (MAP: >120 mmHg, SVRI: >1928 dynes/s/cm^5^/m^2^) range; n: number of measurement sets per device; HDO: high-definition monitor MD^Pro^; petMAP: petMAP graphic II device; SAP, DAP: systolic, diastolic arterial pressure.

**Table 3:** The traffic light system illustrates the agreement of the oscillometric devices with reference arterial pressure. Red implies an overall weak agreement (none of the ACVIM guideline criteria (BROWN et al., 2007) [23] are achieved), yellow a moderate (ACVIM guideline criteria are in part achieved) agreement, and green a good agreement (all of the ACVIM guideline criteria are achieved) with the invasive reference method at the investigated cardiovascular ranges.

		BP			SVRI		
		↔	↓	↑	↔	↓	↑
HDO	SAP						
	MAP						
	DAP						
petMAP	SAP						
	MAP						
	DAP						

Abbreviations: SVRI: systemic vascular resistance index; ↔: in reference range, ↓: low range (MAP: <60 mmHg, SVRI: <1523 dynes/s/cm^5^/m^2^), ↑: high (MAP: >120 mmHg, SVRI: >1928 dynes/s/cm^5^/m^2^); HDO: high-definition monitor MD^Pro^; petMAP: petMAP graphic II device; SAP, MAP, DAP: systolic, mean, diastolic arterial pressure.

## Data Availability

The data presented in this study are available on request from the corresponding author.

## Abbreviations

The following abbreviations are used in this manuscript:

ACVIM	American College of Veterinary Internal Medicine
BP	blood pressure
DAP	diastolic arterial pressure
HR	heart rate
IBP	invasive blood pressure
iSAP	invasively measured SAP
iMAP	invasively measured MAP
iDAP	invasively measured DAP
MAP	mean arterial pressure
NIBP	noninvasive blood pressure
SAP	systolic arterial pressure
SVRI	systemic vascular resistance index
TA	trending ability
